# A Rare Delayed Atypical Pseudoprogression in Nivolumab-Treated Non-Small-Cell Lung Cancer

**DOI:** 10.1155/2019/8356148

**Published:** 2019-03-03

**Authors:** Suyanto Suyanto, Daniel Yeo, Sarah Khan

**Affiliations:** Nottingham University Hospital NHS Trust, UK

## Abstract

Immune checkpoint inhibitors such as Nivolumab work by preventing the inactivation of host T-cells by tumour cells, thereby allowing the T-cells to attack the tumour cells, which results in tumour tissue necrosis. We describe a 78-year-old woman with metastatic lung adenocarcinoma treated with Nivolumab after disease progression following first-line chemotherapy. Computed tomography (CT) after 3 cycles showed a smaller left lower lobe (LLL) primary and stable right lower lobe (RLL) metastatic lesion. CT after 9 cycles showed a reduced RLL mass and an increase in LLL primary. However, CT after 15 cycles showed that the RLL mass had further reduced in size but the LLL mass was significantly larger. The biopsy of the LLL lesion showed necrotic areas and reactive inflammatory changes, without residual malignancy. A repeat CT after further 4 cycles confirmed tumour regression in both the primary and the metastatic lesions. There was a prior reported case of pseudoprogression in a non-small-cell lung cancer patient who had 7 cycles of Nivolumab, and it was diagnosed during a further line of chemotherapy. Here, we report a patient with pseudoprogression during treatment with Nivolumab and at a much later time, after 15 cycles.

## 1. Introduction

Nivolumab works as a checkpoint inhibitor by binding to the T-cell programmed death- (PD-) 1 receptors and therefore preventing the tumour cell PD-ligand 1 (PD-L1) from binding to them and inactivating the T-cells. The use of this therapy is now applied to several malignancies such as melanoma, non-small-cell lung cancer (NSCLC), and urological malignancies, with more studies ongoing for other types of cancers. This recent advancement with immune checkpoint inhibitors has therefore posed its own challenges in the assessment of response to treatment. There have been several reports of pseudoprogression on scheduled CT imaging during the first few weeks of immunotherapy treatment in melanoma and NSCLC. Here, we report the second case of delayed pseudoprogression with Nivolumab in the treatment of NSCLC with the first reported case of a pseudoprogression which occurred after 7 cycles of Nivolumab and a further line of chemotherapy [1], while in this case, the patient had pseudoprogression during treatment with Nivolumab and at a much delayed time after 15 cycles.

## 2. Case Description

A 78-year-old woman was diagnosed with stage IV adenocarcinoma of the left lung in November 2015 after presenting with a history of haemoptysis. Her only medical history was hypercholesterolaemia. She underwent a bronchoscopy and biopsy of a lesion in the LLL, which confirmed TTF-1-positive adenocarcinoma of the lung. Her tumour status was epidermal growth factor receptor (EGFR) mutation and anaplastic lymphoma kinase rearrangement negative. Her initial CT at diagnosis showed a large LLL tumour measuring 5.3 × 7.9 × 6.3 cm with volume loss, satellite nodules, and surrounding interstitial changes. There was a severe encasement and narrowing of the pulmonary vessels, pleura infiltration with discrete pleural nodularity in the left upper lobe, and a small effusion. Bilateral pulmonary metastases were seen with a large nodule in the RLL measuring 2.2 × 2.9 cm. There were also enlarged necrotic appearing lymph nodes in the left hilar and subaortic region, which measured 12 mm.

**Figure 1:**
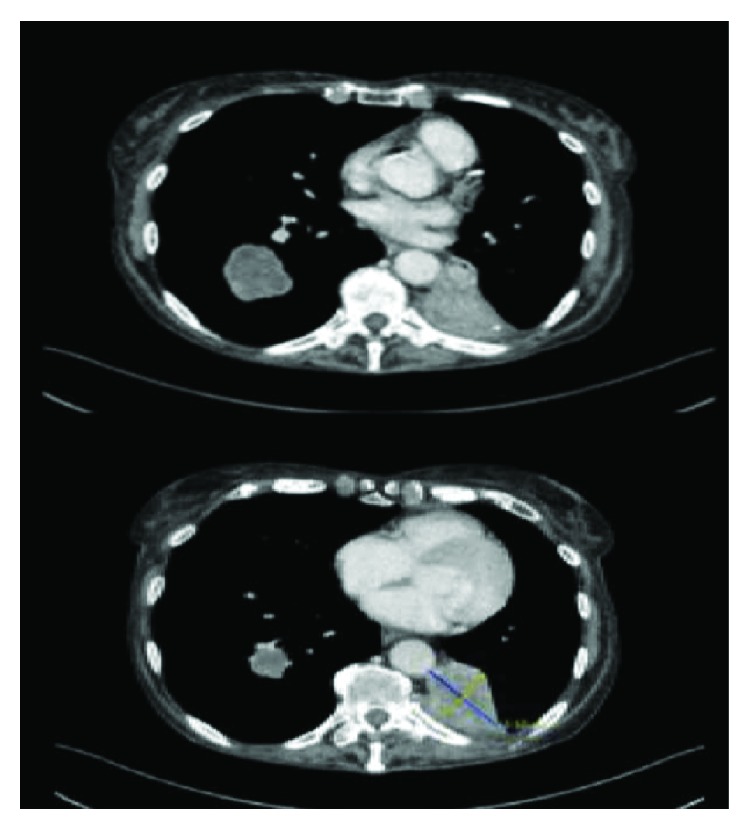
Restaging CT prior to Nivolumab. Image on the top shows the RLL at its largest diameter and the image on the bottom shows the LLL at its largest diameter.

She was initially commenced on palliative chemotherapy with carboplatin and pemetrexed. After 3 cycles of chemotherapy, her restaging CT showed an increase in size of the nodular lesion of RLL measuring 3.8 × 3.5 cm with LLL measuring 5.3 × 3.5 × 5.9 cm and subaortic node of 9 mm (Figure 1). She was commenced on second-line treatment with Nivolumab (3 mg/kg) on the early access to medicine scheme in May 2016, which she tolerated well. An interval restaging CT post 3 cycles of Nivolumab in June 2016 showed a stable RLL mass measuring 3.6 × 3.7 cm, and the LLL mass was smaller measuring 3.1 × 3.6 cm. No mediastinal lymph node enlargement was seen.

**Figure 2:**
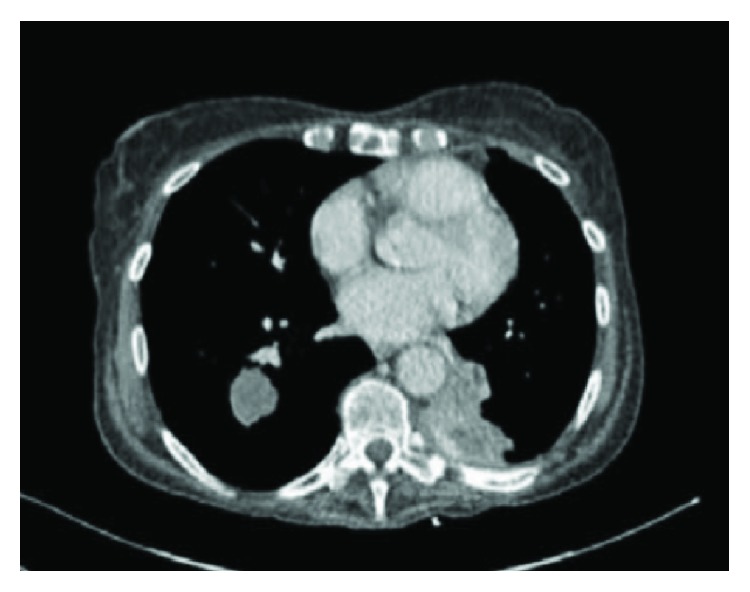
Restaging CT post 9 cycles of Nivolumab. There was a reduction of the RLL mass post 9 cycles of Nivolumab.

**Figure 3:**
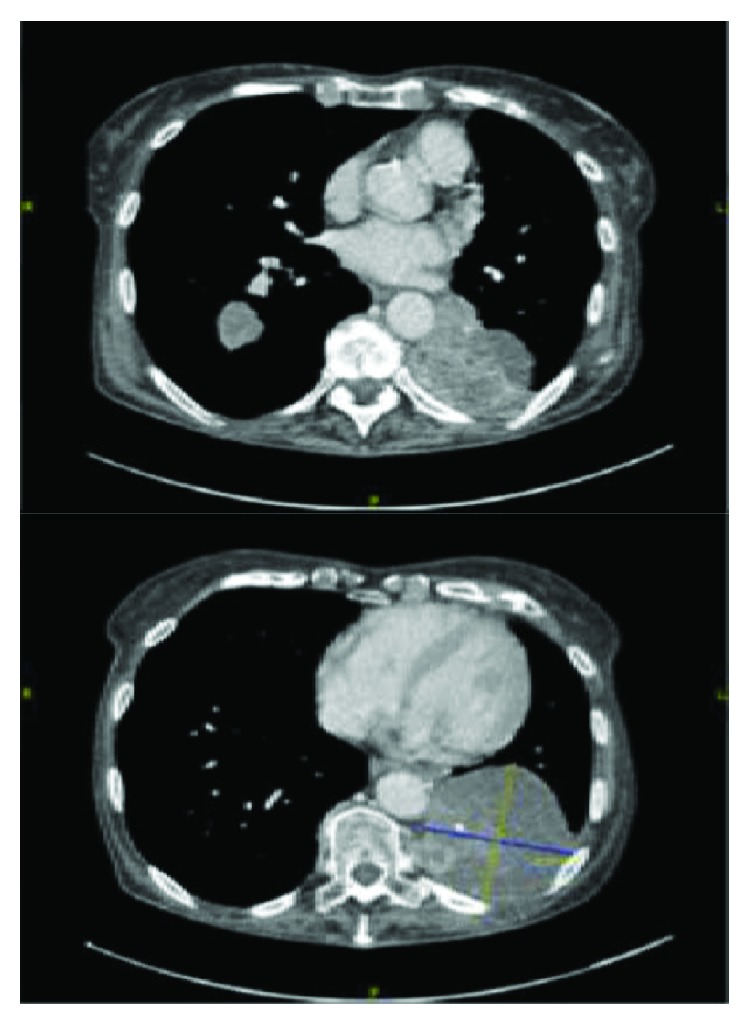
Restaging CT post 15 cycles of Nivolumab. Image on the top shows some reduction in RLL mass and the image on the bottom shows that the LLL mass had increased significantly.

A restaging scan after 9 cycles of Nivolumab in September 2016 showed some reduction in the RLL mass measuring 3.1 × 2.8 cm, an increase in LLL lesion 4.3 × 3.9 cm (Figure 2). A further interval CT restaging after 15 cycles of Nivolumab in December 2016 showed that the RLL mass had further reduced in size measuring 2.9 × 2.6 cm. The LLL mass was, however, significantly larger measuring 7.7 × 7.3 cm. This mass has lobulated margins and showed marginal and almost septated more central enhancement. Stable pleural thickening is shown in Figure 3. Her case was discussed in the lung multidisciplinary team meeting, and she went on to have an ultrasound-guided biopsy of LLL mass in January 2017. The histopathology report concluded fragments of lung parenchyma with necrotic areas and reactive inflammatory changes, with no evidence of residual malignancy, features which were in keeping with immunotherapy effect. We concluded based on the biopsy findings that the significant radiological size increase was due to pseudoprogression, and she continued with the immunotherapy.

**Figure 4:**
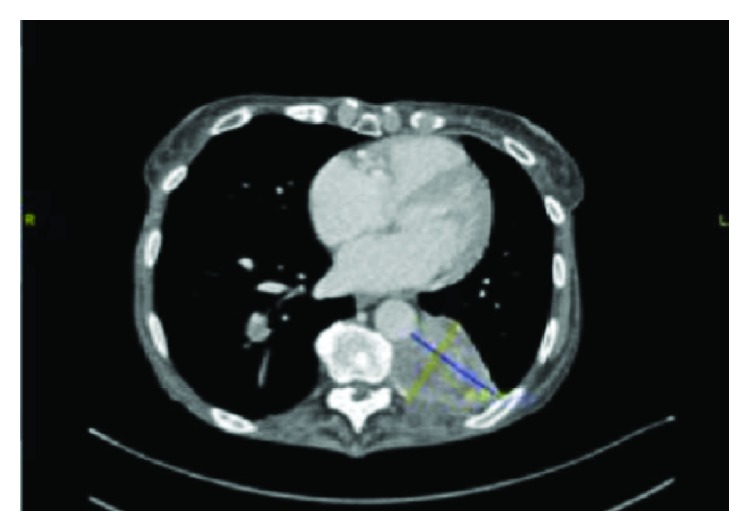
Restaging CT post 19 cycles of Nivolumab. There was a tumour regression in the RLL and LLL mass.

A further repeat CT scan was performed after further 4 cycles in March 2017 which confirmed tumour regression with the LLL mass measuring 6.5 cm × 5.3 cm. The mass in the RLL also showed a reduction in size measuring 2.1 × 2.0 cm in maximum axial diameter (Figure 4).

## 3. Discussion

The treatment with immunotherapy is dependent upon the ability of the immune system to recognise malignant from normal cells and this, in part, relies on the antigenicity of the tumour cells [2, 3]. Tumour cells escape immune-mediated elimination by losing their antigenicity via dysregulation of antigen presentation machinery at the epigenetic, transcriptional, and posttranscriptional levels and also via immune selection of cells that lack immunogenic antigens [2, 4].

Another method of evasion by tumour cells is by decreasing their immunogenicity [2, 5]. This can be achieved by immunoinhibitory PDL1 upregulation via epigenetic factors, oncogenic signaling (e.g., phosphatidylinositol-3-kinase-protein kinase B (PI3K-AKT) pathway or signal transducer and activator of transcription- (STAT-) 3 signaling), and acquired immune responses [6]. In solid tumours like NSCLC, acquired immune responses mediated by interferon-gamma (IFN-*γ*) from tumour-infiltrating lymphocytes (TIL) are thought to lead to the upregulation of PD-L1 [5]. Importantly, not all PD-L1-expressing tumours are associated with immune infiltration or response to anti-PD-1 treatment [5]; hence, additional markers of immunogenicity may be involved, such as the immune checkpoint molecules on tumour cells and surrounding stromal cells or negative regulatory markers on TIL [7, 8].

Tumour microenvironment also plays a crucial role in the escape of tumour cells from elimination by the immune cells. Some tumours recruit immunosuppressive leukocytes to generate an immunosuppressive microenvironment, and the elimination of these immunosuppressive cell populations has been found to restore T-cell infiltration into tumour tissues and their ability to mediate antitumour activity [2].

The mechanism underlying pseudoprogression is, however, less clear. The phenomenon of apparent increase in tumour burden could be explained by either transient immune cell infiltration, extensive inflammation, vascular permeability, and oedema or continued tumour growth preceding a delayed effect of immune cells [9]. Hence, the conventional assessment of response to treatment with Response Evaluation Criteria in Solid Tumors (RECIST), which measures the unidimensional longest diameter to quantify tumour growth, could be misleading [10, 11]. Recent development of criteria to assess response to immunotherapy treatment, such as unidimensional Immune-Related RECIST (irRECIST) and bidimensional measurement with immune-related response criteria (irRC) or iRECIST, for use in clinical trial setting has allowed a more accurate imaging assessment [11, 12]. Despite this, true progression could potentially still be missed and this can result in inadequate treatments. Recent development with liquid biopsy with molecular biomarkers could potentially avert the need to perform a repeat biopsy in this subset of patients. A recent study has shown droplet digital PCR of circulating tumour DNA kinetics correlate to treatment response in KRAS-mutated adenocarcinoma with rapid decrease in patients with pseudoprogression and rapid increase in true progression [13].

## 4. Conclusion

Pseudoprogression in immunotherapy-treated solid tumours can be either early or delayed. The assessment by current gold standard RECIST criteria can be misleading and irRECIST is a better way to assess the CT imaging of these patients. The importance of clinical assessment could not be overstated, and in cases which pseudoprogression is suspected, a biopsy should be performed. The development of liquid biopsy has certainly offered the prospect of a less invasive way of diagnosing these cases of pseudoprogression.
